# Longitudinal Assessment of a Manualized Group Treatment Program for Gambling Disorder: The Ohio Problem Gambling Treatment Model for Adults with Co-Occurring Disorders

**DOI:** 10.1007/s10899-021-10089-0

**Published:** 2022-01-01

**Authors:** Aaron J. Kruse-Diehr, Stephen R. Shamblen, Matthew W. Courser

**Affiliations:** 1 University of Kentucky College of Public Health, 151 Washington Ave, Bowman Hall 345, Lexington, KY 40536 USA; 2 Pacific Institute for Research and Evaluation, 401 W Main St, Suite 2100, Louisville, KY 40202 USA

**Keywords:** Gambling disorder, Co-occurring disorders, Behavioral health, Manualized treatment

## Abstract

Individuals with gambling disorder (GD) experience a host of negative psychosocial and physical health outcomes, yet few seek treatment. Of particular concern are individuals with co-occurring mental and behavioral health disorders, a group at higher risk for GD in the state of Ohio. To better serve this population, the Ohio Department of Mental Health and Addiction Services developed a group-based GD treatment manual for adults with co-occurring disorders. Over the course of 5 years, 353 individuals engaged in at least some of the manual’s 12 weekly modules, and more than one-third (*n* = 122) completed the entire curriculum. Participants who completed all 12 modules completed pre-and post-tests, and after controlling for covariates, participants significantly decreased their GD symptom severity, though changes in self-esteem and gambling urges were non-significant. These findings suggest the treatment manual holds promise at reducing gambling behaviors for individuals with co-occurring disorders, but further research is warranted to explore best practices on how to intervene on the psychological antecedents to gambling in this population.

An estimated 0.4% to 0.6% of individuals in the United States (US) experience pathological gambling, also known as gambling disorder (GD), in their lifetimes, with increased prevalence among individuals with co-occurring psychiatric and behavioral health disorders (Cowlishaw & Hakes, 2015; Grant et al., 2005; Kessler et al., 2008; Shaffer et al., 1999). Gambling disorder is defined in the Diagnostic and Statistical Manual of Mental Disorders (5th ed.; DSM–5; American Psychiatric Association [APA] 2013) as the persistence and recurrence of problematic gambling behavior that leads to clinically significant impairment and distress. Although previously considered an “Impulse-Control Disorder” in previous editions of the DSM, GD is classified in the DSM-5 as a “Substance-Related and Addictive Disorder,” due, in part, to the similarities between people with disordered gambling and those with substance use disorders (APA 2013). People with GD display cravings and “highs” like individuals with substance abuse issues, and there is also similar evidence of a genetic link among those with GD (Potenza et al., 2005; Reilly & Smith, 2013).

Individuals affected by GD often experience a variety of adverse psychosocial effects stemming from their addiction, including higher levels of work absenteeism, increased use of medical services, jeopardized personal relationships, and lower self-esteem, all of which can contribute to increased stigma for the individual with GD (APA 2013; Rogier & Velotti, 2017). Their health status is negatively affected as well, with this population being nearly twice as likely to report their general health as fair or poor, and twice as likely to have sought professional help for emotional or mental health problems, than low-risk gamblers (National Gambling Impact Study Commission, 1999). In 2017, 10.3% of Ohioans, or 919,162 adults, were considered at-risk for GD, with an additional 0.9%, or 76,379 adults, who could likely be clinically diagnosed with GD. Those at greatest risk for GD in Ohio displayed higher rates of co-occurring health and socioeconomic consequences, such as higher unemployment, increased depression and stress, lower educational attainment, and increased rates of alcohol and substance use disorders (Ohio for Responsible Gambling, 2017). Cumulatively, the social costs of gambling addiction in Ohio are calculated to be just over $230 million dollars, with negative effects disproportionately found among individuals living in poverty (National Council on Problem Gambling, 2009; National Institutes of Health, 2011). Despite the multiple negative individual and social sequelae of GD, only 10 percent of those with GD actually seek treatment (Cunningham, 2005).

With the available options for gambling and gaming having increased exponentially over recent years, it is critical that practitioners in mental health and addiction agencies adopt a public health approach to GD that includes both prevention and treatment—especially in cities with large, diverse populations and with a greater range of gambling options. Although many manualized approaches have been developed to treat GD, few can be considered evidence-based. The Substance Abuse and Mental Health Services Administration’s (SAMHSA) National Registry of Evidence-based Programs and Practices (NREPP) contained only two evidence-based interventions (EBIs) focusing specifically on GD, an in-school model targeted at adolescent youth and a telephone-based interview followed by a self-help workbook (SAMHSA 2014). In 2018, NREPP was suspended by the US Assistant Secretary of Mental Health and Substance Use and replaced by the Evidence-Based Practices Resource Center, a repository that currently features no gambling-specific EBIs. There remains a need for standardized EBIs that focus specifically on adults with GD. In addition, because individuals affected by GD often have other psychiatric comorbidities and can engage in and/or transfer GD to other addictive behaviors and substances, there is a critical need for EBIs and treatment approaches that address GD as well as other comorbidities, of which there are presently very few options (Downling et al. 2016). Additionally, there is very little extant evidence to inform treatment recommendations for individuals affected by GD and other comorbidities and few studies that assess whether GD and comorbidities should be treated concurrently or sequentially (Dowling et al., 2016).

To address this gap, the Ohio Department of Mental Health and Addiction Services (OhioMHAS), in consultation with multiple stakeholders with expertise in treating GD, developed a group-based treatment manual to address GD in adults with co-occurring disorders, a group with higher rates of GD in Ohio (Ohio for Responsible Gambling, 2017). Though the manual was designed for this specific subpopulation, the funding for the project specified a primary focus on GD treatment, and thus the project’s specific aims were to assess, treat, and measure changes in GD. and the manual included treatment methods and approaches that have been used successfully in individuals with behavioral health and substance use disorders. Ultimately, the overarching goal of the intervention was to address the negative consequences of GD and reduce the number of people with GD across and within various populations in Ohio. The present study describes the process of creating the intervention and reports longitudinal findings on its effects on self-esteem, gambling urges, and GD symptom severity among diverse participants throughout Ohio.

## Methods

### Intervention

The Ohio Problem Gambling Treatment Model for Adults with Co-Occurring Disorders (OhPGTM) was developed by multiple stakeholders with expertise in GD, including clinical staff from Zepf Center in Toledo, Ohio and other clinicians from across the state. Subject area experts helped refine the final version of the model. The OhPGTM was administered across seven sites in Ohio, in both urban and rural locations, over the course of 5 years to determine its clinical effectiveness at improving key GD outcomes for individuals with GD and other co-occurring disorders. By 2019, a total of 353 individuals engaged in at least some part of the OhPGTM, and slightly more than one-third (*n* = 122) completed all 12 weeks of the curriculum.

Implementation of the OhPGTM was guided by a treatment manual that was developed using a multi-therapeutic approach, drawing from Cognitive Behavioral Therapy (CBT) techniques, Motivational Interviewing (MI), Stages of Change, and the promotion of life skills. The manual consisted of 12 weeks of programming addressing various antecedents of GD, wherein each module built upon lessons learned from prior weeks. Examples of modules included “Addiction Theory,” “Interpersonal Skills Building,” “Triggers,” “Rational Thinking,” “Defense Mechanisms,” and “Relapse,” among others. These modules were developed specifically to address issues related to gamblers with co-occurring disorders. For example, in the “Rational Thinking” module, the counselor facilitated group-based MI sessions, an approach used more frequently to address behavioral and substance use disorders (see Krejci & Neugebauer, 2015; Velasquez et al., 2006) than GD (see Josephson et al., 2016). In this module, participants listed various life events that might trigger co-occurring disorders like depression, anxiety, substance abuse, and other potentially harmful behaviors, and the facilitator then discussed pathways by which these behaviors can, in turn, trigger unwanted gambling behaviors. Similarly, the module “Assertiveness” used CBT techniques (see Speed et al., 2018) to address anxiety and depression, illustrating how passivity can promote these disorders which can, in turn, trigger GD. Yet another example is the “Relapse” module, which specifically addresses how co-occurring disorders like anxiety or alcohol use might lead to GD, drawing on Stages of Change to help participants learn how various mental states might promote or inhibit recognition of relapse warning signs. Each weekly topic began with facilitator instructions and included an interactive didactic segment, a review of a GD craving scale and monitoring logs, handouts, and a take home assignment. Participants signed a group member contract, in which they agreed to attend all sessions, abstain from alcohol and drugs at least 24 h prior to group, admit if they have gambled in between group sessions, and maintain confidentiality of other group members. Each group consisted of between 3–12 participants.

### Measures

Prior to beginning the program, and immediately upon completion of the 12th session, participants were asked to complete a one-page, front and back, easy to read, identical pre-test/post-test survey to determine the clinical effectiveness of the OhPGTM. The instrument, which focused on GD outcomes exclusively, was comprised of three scales: (1) The Gambling Craving Scale (GACS; Young & Wohl, 2009) was used to assess distal factors related to gambling; (2) The Problem Gambling Severity Index (PGSI; Ferris & Wynne, 2001) was used to assess previous year’s actual GD symptom severity and its effects on both the individual and family members; and (3) The Rosenberg Self-Esteem Scale (RSES; Rosenberg, 1965), a scale historically administered to individuals with GD, was used to assess participant self-esteem. Additionally, the survey collected demographic information on age, gender, race/ethnicity, and comorbid disorders (i.e., mental health and substance use disorders).

The two scales used to assess gambling attitudes and behaviors were established by reviewing published literature in the GD field. The first scale, the GACS, was selected based on its high levels of reliability and validity across a variety of populations (Young & Wohl, 2009). While the GACS has three subscales, more recent evidence has suggested two underlying dimensions that are correlated (Canale et al., 2019). As such, and as all items exhibited high internal consistency, we calculated a total scale score for this measure. The 9-item short form PGSI was also shown to be consistent and invariant across various demographic markers, including age, gender, location, and gambling type (Arthur et al., 2008; Holtgraves, 2009; Miller et al., 2013). Total scale scores were calculated for the PGSI. To demonstrate face and content validity, experts in the areas of psychometrics and GD were solicited to provide feedback prior to survey finalization. In the present study, each individual scale displayed high levels of internal consistency (RSES: α = 0.90, GACS: α = 0.91, PGSI: α = 0.87).

### Analysis

The main analyses conducted for this paper examined whether there were changes over time in the targeted GD outcomes. We explored this using repeated measures t-tests and multiple least squares regression. We first examined whether the unadjusted difference was significant using a simple repeated measures t-test, and we next examined whether the adjusted difference was statistically significant by regressing the outcome difference score (post-test–pre-test) on sex, race (dummy variables for white, Black, and Latino), having a mental health disorder, and having a substance use disorder. The intercept in this regression analysis yields a significance test that is identical to the repeated measures effect in a repeated measures ANOVA with covariates. We also calculated the effect size d, which represents the extent to which the mean difference scores is different from zero in standard deviation units. Effects sizes are generally interpreted as small (d = 0.20), medium (d = 0.50), and large (d = 0.80; Cohen, 1988). All statistical analyses were conducted either with IBM SPSS Version 26 or the R environment for statistical computing (Ihaka & Gentleman, 1996). The authorship team did not have any involvement in the project beyond implementation guidance, content expertise for creation of materials, statistical analysis duties, and professional research writing. Given these specific roles, and because the authorship team received de-identified data solely for analysis purposes, this project was deemed Not Human Research (NHR) by the University of Kentucky Institutional Review Board.

## Results

Over the course of 5 years of administration of the OhPGTM, a total of 122 participants completed the program. Participants were well balanced on sex (51% female and 49% male), and three-quarters were white (75%). Black (12%), Latino (10%), Asian (1%), and mixed-race (3%) individuals were also represented in the sample. Behavioral health diagnoses were common in the sample, as about two-thirds had a mental health disorder (defined as depression, anxiety, mania, or psychosis), and almost three-quarters had a substance use disorder. See Table 1 for additional details.

**Table 1:** Demographics of Study Participants

Variable	*n*	(%)
Gender		
Female	62	(51)
Male	60	(49)
Race/Ethnicity		
White	91	(75)
Black or African American	15	(12)
Hispanic or Latino/a	10	(8)
Asian or Pacific Islander	1	(1)
More than one race	4	(3)
Other	1	(1)
Co-occurring disorders		
Other drugs	81	(66)
Depression	78	(64)
Anxiety	76	(62)
Alcohol dependence	34	(28)
Mania	19	(16)
Psychosis	12	(10)
Other	21	(17)

*Note N* = 122; Percentages for co-occurring disorders may exceed 100% because participants could check all that applied

To ensure observed changes for those who completed treatment (i.e., did not drop out) were due to the intervention and not self-selection, we examined whether the profile of participant background characteristics predicted treatment attendance. Of the 353 unique individuals who enrolled in the program and completed the pre-test survey, slightly more than a third (35% or 122) completed the program and the post-test survey. A probit regression model was performed by regressing treatment attendance on sex, race (dummy variables for white, Black, and Latino), having a mental health disorder, and having a substance use disorder. Neither the omnibus model test, χ^2^(6) = 8.50, *p* = 0.204, nor significant model coefficients (*p* < 0.05) suggested selectivity biases. Further, we examined whether there were differences between these groups on targeted outcomes at baseline (i.e., self-esteem, gambling urges, and GD symptom severity) and found no significant differences (*p* < 0.05). As such, we did not adjust estimates for potential selectivity biases (e.g., Heckman, 1976).

Unadjusted comparisons with repeated measures t-tests suggested there were significant increases in participant self-esteem and significant decreases in gambling urges and GD symptom severity. When controlling for covariates, only a statistically significant decrease in self-reported GD symptom severity remained. While no covariates were significantly related to the change for any outcome (*p* < 0.05), they clearly affected the change when working in concert (Table 2).

**Table 2:** Pre- to Post-Test Changes in Outcomes Targeted by OhPGTM

Construct	Pre		Post			Unadjusted		Adjusted	
	M	SD	M	SD	d	*t*	*p*	*t*	*p*
Self-esteem	2.55	.60	3.09	.56	.85	9.57	< .001	.12	.904
Gambling urges	2.53	.56	1.90	.52	− .97	− 10.78	< .001	− 0.37	.716
GD symptom severity	2.65	.62	2.23	.83	− .53	− 5.72	< .001	− 2.81	.006

*Note N* = 122

## Discussion

During its 5 years of administration, the group-based OhPGTM was used to treat 353 individuals with co-occurring disorders at behavioral health agencies across Ohio, and more than a third completed all 12 weeks of treatment. Treatment completion rates for outpatient programs range from 17 to 71% (Westphal 2007), positioning the OhPGTM program’s treatment completion within typical rates of other behavioral health GD treatment programs. Participants who completed the OhPGTM program experienced reduction in self-reported gambling, suggesting the program achieved its desired behavioral effect. However, after adjusting for covariates, neither participant self-esteem nor gambling urges changed significantly. Given that attitude is often considered a significant predictor of behavior (DeFleur & Westie, 1963), further investigation of the psychological processes driving behavior change among those treated with the OhPGTM is needed.

While other behavioral health programs have successfully utilized group-based approaches to address GD treatment (Carlbring et al., 2009; Jiménez-Murcia et al., 2007, 2019), few, if any, are guided by manuals tailored specifically for individuals with GD and other co-occurring disorders. Instead, most are guided by CBT (Josephson et al., 2016) or other similar therapeutic approaches (e.g., mindfulness-based cognitive therapy; de Lisle et al., 2011). Individual practitioners, then, select methods by which to apply these theories, often guided by theoretical models that apply CBT concepts to GD (see Sharpe & Tarrier, 1993). In multiple respects, then, the OhPGTM is unique; it is guided by different theoretical approaches–including CBT, MI, Stages of Change, and life skills theory–applied not just to gambling behavior directly but also pragmatically to mechanisms by which mental/behavioral health and substance use disorders can contribute to GD, thus providing a standardized and replicable approach to GD therapy in individuals affected by GD and other co-occurring disorders. Manualized approaches have been widely and effectively used to treat substance use disorders (Korecki et al., 2020) and other behavioral health issues, largely because they promote treatment fidelity (Bellg et al., 2004). Manualized approaches developed specifically for individuals with both GD and co-occurring mental or behavioral health disorders, however, are not common. The OhPGTM, thus, represents a significant improvement in GD treatment for a population particularly at risk for negative impacts of GD; the approach in the OhPGTM might prove similarly effective for other behavioral health treatment practitioners.

Future research should prioritize the study of the effectiveness of manualized interventions to address GD in specialized populations that draw from multiple theoretical models and employ methods addressing both behaviors and psychological processes, and evaluation should include specific measures to assess change in not just markers of GD but mental or behavioral health outcomes as well. Furthermore, these studies should employ a rigorous control group to determine whether changes in attitudes and behavior can be attributed to therapy in general or a specific manual. In upcoming years, OhioMHAS intends to further modify the OhPGTM based on clinician feedback from the last 5 years of administration and then implement the OhPGTM across multiple behavioral health organizations in Ohio with random selection of providers to serve as comparison groups (i.e., standardized treatment). Nevertheless, this preliminary investigation suggests that the OhPGTM was effective in reducing self-reported GD symptom severity in a large sample of individuals with GD and other co-occurring disorders throughout Ohio.

### Limitations

Findings from this research should be interpreted in light of some important limitations. First, and most importantly, this study describes the cumulative findings from multiple treatment groups using the OhPGTM but does not compare changes in participant self-esteem, gambling urges, or GD symptom severity to a control group. Accordingly, it is not possible to state with confidence that changes in participant self-reported behavior occurred solely as a result of the manualized approach; indeed, behavior change is multifactorial, and future research should determine specific pathways by which attitudinal and behavior change occurs among individuals receiving treatment for GD (e.g., the group setting, the manual, the treatment provider, peer support). Second, our findings suggest participants reduced their GD symptom severity; however, changes in their gambling urges and self-esteem were not significant. It is thus possible that social desirability bias might partly explain this finding; however, many participants had previously sought treatment and relapsed prior to participating in this study, so it is equally possible that this novel curriculum did indeed produce (at least short-term) behavioral change. Previous studies have suggested between 13 and 18 therapeutic sessions are necessary for clinical improvement in about half of patients, and it is likely that many participants engaged in multiple therapy sessions prior to accrual in the present study (Hansen et al., 2002). Future similarly-designed studies should consider tracking mean number of completed modules to determine dose–response effect. Third, while our sample was comprised of a nearly-even split between males and females, it skewed heavily toward white individuals. In Ohio, Black or African American individuals have the highest rates of GD (Ohio for Responsible Gambling, 2017), and given that multiple studies have suggested racial and ethnic minorities have higher rates of GD than white individuals (Barry et al., 2011a, 2011b; Caler et al., 2017), any future evaluation of the OhPGTM should seek to obtain a more racially diverse sample of participants. Lastly, while the OhPGTM was designed for individuals with co-occurring disorder and featured therapeutic methods and approaches commonly used in mental and behavioral health, the evaluation materials did not include measures of change related to co-occurring disorders; though the OhioMHAS expansion of the project intends to include these measures, these data are not available for the present study.

## Conclusion

A manualized, multi-theoretical treatment model was implemented over 5 years in seven behavioral health organizations throughout Ohio. Evaluation of the model found it to be effective at reducing self-reported GD symptom severity but not at improving self-esteem or reducing gambling urges among individuals with GD and other co-occurring disorders. These promising early findings suggest that additional research into the effectiveness of the OhPGTM, particularly among racial and ethnic minorities affected by GD and other disorders and as compared to standard treatment, is both necessary and warranted.

## References

[CR1] American Psychiatric Association. (2013). *Diagnostic and statistical manual of mental disorders* (5th ed.). Retrieved from 10.1176/appi.books.9780890425596.

[CR2] Arthur D, Tong WL, Chen CP, Hing AY, Sagara-Rosemeyer M, Kua EH, Ignacio J (2008). The validity and reliability of four measures of gambling behavior in a sample of Singapore university students. Journal of Gambling Studies.

[CR3] Barry DT, Stefanovics EA, Desai RA, Potenza MN (2011). Differences in the associations between gambling problem severity and psychiatric disorders among black and white adults: Findings from the National Epidemiologic Survey on Alcohol and Related Conditions. American Journal on Addictions.

[CR4] Barry DT, Stefanovics EA, Desai RA, Potenza MN (2011). Gambling problem severity and psychiatric disorders among Hispanic and white adults: Findings from a nationally representative sample. Journal of Psychiatric Research.

[CR5] Bellg AJ, Borrelli B, Resnick B, Hecht J, Minicucci DS, Ory M (2004). Enhancing treatment fidelity in health behavior change studies. Health Psychology.

[CR6] Caler KR, Garcia JRV, Nower L (2017). Problem gambling among ethnic minorities: Results from an epidemiological study. Asian Journal of Gambling Issues and Public Health.

[CR7] Canale N, Cornil A, Giroux I, Bouchard S, Billieux J (2019). Probing Gambling Urge as a State Construct: Evidence From a Sample of Community Gamblers. Psychology of Addictive Behaviors.

[CR8] Carlbring P, Jonsson J, Josephson H, Forsberg L (2009). Group therapy in the treatment of problem gambling: A randomized controlled trial. Cognitive Behavioral Therapy.

[CR9] Cohen J (1988). Statistical power analysis for the behavioral sciences.

[CR10] Cowlishaw S, Hakes JK (2015). Pathological and problem gambling in substance use treatment: Results from the National Epidemiologic Survey on Alcohol and Related Conditions (NESARC). American Journal on Addictions.

[CR11] Cunningham JA (2005). Little use of treatment among problem gamblers. Psychiatric Services.

[CR12] DeFleur ML, Westie FR (1963). Attitude as a scientific concept. Social Forces.

[CR13] de Lisle SM, Dowling NA, Allen JS (2011). Mindfulness-based cognitive therapy for problem gambling. Clinical Case Studies.

[CR14] Dowling NA, Merkouris SS, Lorains FK (2016). Interventions for comorbid problem gambling and psychiatric disorders: Advancing a developing field of research. Addictive Behaviors.

[CR15] Ferris, J., & Wynne, H. (2001). The Canadian problem gambling index: Final report. Ottawa: Canadian Centre on Substance Abuse.

[CR16] Grant JE, Levine L, Kim D, Potenza MN (2005). Impulse control disorders in adult psychiatric populations. American Journal of Psychiatry.

[CR17] Hansen NB, Lambert MJ, Forman EM (2002). The psychotherapy dose-response effect and its implications for treatment delivery services. Clinical Psychology Science and Practice.

[CR18] Heckman JJ (1976). The common structure of statistical models of truncation, sample selection and limited dependent variables and a simple estimator for such models. Annals of Economic and Social Measurement.

[CR19] Holtgraves T (2009). Evaluating the problem gambling severity index. Journal of Gambling Studies.

[CR20] Ihaka R, Gentleman R (1996). A language for data analysis and graphics. Journal of Computational and Graphical Statistics.

[CR21] Jiménez-Murcia S, Álvarez-Moya EM, Granero R, Aymami MN, Gómez-Peña M, Jaurrieta, N…Vallejo, J.  (2007). Cognitive-behavioral group treatment for pathological gambling: Analysis of effectiveness and predictors of therapy outcome. Psychotherapy Research.

[CR22] Jiménez-Murcia S, Granero R, Fernández-Aranda F, Aymamí N, Gómez-Peña M, Mestre-Bach G (2019). Developmental trajectories of gambling severity after cognitive-behavioral therapy. European Psychiatry.

[CR23] Josephson, H., Carlbring, P., Forsberg, L., & Rosendahl, I. (2016). People with gambling disorder and risky alcohol habits benefit more from motivational interviewing than from cognitive behavioral group therapy. *PeerJ*, *4*, e1899. 10.7717/peerj.1899 PMC4824888 27069823

[CR24] Kessler RC, Hwang I, LaBrie R, Petukhova M, Sampson NA, Winters KC, Shaffer HJ (2008). DSM-IV pathological gambling in the National Comorbidity Survey Replication. Psychological Medicine.

[CR25] Krejci J, Neugebauer Q (2015). Motivational interviewing in groups: Group process considerations. Journal of Groups in Addiction & Recovery.

[CR43] Korecki, J. R., Schwebel, F. J., Votaw, V. R., & Witkiewitz, K. (2020). Mindfulness-based programs for substance use disorders: A systematic review of manualized treatments. *Substance Abuse Treatment Prevention and Policy*, *15*(1). 10.1186/s13011-020-00293-3 10.1186/s13011-020-00293-3 PMC7392831 32727559

[CR26] Miller NV, Currie SR, Hodgins DC, Casey D (2013). Validation of the problem gaming severity index using confirmatory factor analysis and Rasch modelling. International Journal of Methods in Psychiatric Research.

[CR27] National Council on Problem Gambling. (2009). *Ohio 2009 gambling and problem gambling estimates*. Retrieved from http://www.ncpgambling.org/files/Help%20By%20State%20Fact%20Sheets/Ohio%20Fact%20Sheet.pdf.

[CR28] National Gambling Impact Study Commission. (1999). *Gambling impact and behavior study*. Retrieved from http://www.norc.org/PDFs/publications/GIBSFinalReportApril1999.pdf.

[CR29] National Institutes of Health. (2011). *When the stakes turn toxic: Learn about problem gambling*. Retrieved from http://newsinhealth.nih.gov/issue/May2011/Feature1.

[CR31] Ohio for Responsible Gambling. (2017). *Ohio Gambling Survey – Round Two SFY 2016–17.* Retrieved from https://mha.ohio.gov/Portals/0/assets/FamiliesChildrenandAdults/Get%20Help/Problem%20Gambling/SFY-16-17-OH-Gambling-Survey-Highlights.pdf.

[CR32] Potenza MN, Xian H, Shah K, Scherrer JF, Eisen SA (2005). Shared genetic contributions to pathological gambling and major depression in men. Archives of General Psychiatry.

[CR33] Reilly, C., & Smith, N. (2013). The evolving definition of pathological gambling in the DSM-5. *National Center for Responsible Gaming*. Retrieved from http://www.ncrg.org/sites/default/files/uploads/docs/white_papers/ncrg_wpdsm5_may2013.pdf.

[CR34] Rogier, G., & Velotti, P. (2017). Impulsivity and self-esteem in pathological gambling: What is the link? *European Psychiatry, 41*(Suppl.), S394.

[CR35] Rosenberg M (1965). Society and the adolescent self-image.

[CR36] Shaffer HJ, Hall MN, Vander Bilt J (1999). Estimating the prevalence of disordered gambling behavior in the United States and Canada: A research synthesis. American Journal of Public Health.

[CR37] Sharpe L, Tarrier N (1993). Towards a cognitive-behavioural theory of problem gambling. British Journal of Psychiatry.

[CR38] Speed BC, Goldstein BL, Goldfried MR (2018). Assertiveness training: A forgotten evidence-based treatment. Clinical Psychology Science and Practice.

[CR39] Substance Abuse and Mental Health Services Administration. (2014). *SAMHSA’s national registry of evidence- based programs and practices*. Retrieved from http://nrepp.samhsa.gov/Index.aspx.

[CR40] Velasquez MM, Stephens NS, Ingersoll K (2006). Motivational interviewing in groups. Journal of Groups in Addiction & Recovery.

[CR41] Westphal JR (2007). Are the effects of gambling treatment overrated?. International Journal of Mental Health and Addiction.

[CR42] Young MM, Wohl MJ (2009). The gambling craving scale: Psychometric validation and behavioral outcomes. Psychology of Addictive Behaviors.

